# Perceptual representation and effectiveness of local figure–ground cues in natural contours

**DOI:** 10.3389/fpsyg.2015.01685

**Published:** 2015-11-03

**Authors:** Ko Sakai, Shouhei Matsuoka, Ken Kurematsu, Yasuhiro Hatori

**Affiliations:** Computational Vision Science Laboratory, Department of Computer Science, University of TsukubaTsukuba, Japan

**Keywords:** perception, Gestalt factor, contour shape, natural image, border ownership, psychophysical experiment

## Abstract

A contour shape strongly influences the perceptual segregation of a figure from the ground. We investigated the contribution of local contour shape to figure–ground segregation. Although previous studies have reported local contour features that evoke figure–ground perception, they were often image features and not necessarily perceptual features. First, we examined whether contour features, specifically, convexity, closure, and symmetry, underlie the perceptual representation of natural contour shapes. We performed similarity tests between local contours, and examined the contribution of the contour features to the perceptual similarities between the contours. The local contours were sampled from natural contours so that their distribution was uniform in the space composed of the three contour features. This sampling ensured the equal appearance frequency of the factors and a wide variety of contour shapes including those comprised of contradictory factors that induce figure in the opposite directions. This sampling from natural contours is advantageous in order to randomly pickup a variety of contours that satisfy a wide range of cue combinations. Multidimensional scaling analyses showed that the combinations of convexity, closure, and symmetry contribute to perceptual similarity, thus they are perceptual quantities. Second, we examined whether the three features contribute to local figure–ground perception. We performed psychophysical experiments to judge the direction of the figure along the local contours, and examined the contribution of the features to the figure–ground judgment. Multiple linear regression analyses showed that closure was a significant factor, but that convexity and symmetry were not. These results indicate that closure is dominant in the local figure–ground perception with natural contours when the other cues coexist with equal probability including contradictory cases.

## Introduction

The visual system segregates a scene into regions and assigns figure and ground to them. The shape of the region boundary strongly influences the figure–ground segregation. A number of contour features, such as convexity, closure, symmetry, good continuation, similarity, and proximity, are cues for the segregation (e.g., Brunswik and Kamiya, 1953; Kanizsa, 1979). For instance, psychophysical studies reported a decrease in the detection threshold (e.g., Kovács, 1996) and a decrease in the response time (Elder and Zucker, 1998) for collinearly aligned patches and closed boundaries, respectively. A wide range of saliency that depends on symmetry has also been reported (Rainville and Kingdom, 2000; Cohen and Zaidi, 2013). Functional MRI studies have reported the selective responses to colinear contours in the early (V1 and V2) to higher (lateral occipital complex) visual cortical areas (Kourtzi et al., 2003), and the responses to symmetry that were highly correlated with human perception in the intermediate (V3A, V4, and V7) to higher (lateral occipital) areas (Sasaki et al., 2005). Combinations of plural cues have also been studied (Devinck and Spillmann, 2013; Froyen et al., 2013; Mojica and Peterson, 2014). Although contours in natural scenes usually constitute multiple cues, previous studies have focused on testing stimuli in which each cue was independently provided with simplified and artificial topographies.

How coexisting multiple cues contributes to figure–ground perception and how conflicting cues fuse to produce coherent perception have not been clarified. Analyses of the perception of natural contours appear to be ingenious for answering these questions. Within local patches from natural contours, convexity, vertical location, and size provide clues for figure–ground segregation, with convex, lower, and smaller regions tending to be associated with figures (e.g., Fowlkes et al., 2007; Burge et al., 2010). Combining two or three of these cues further facilitates the perception of figures. Although these studies provided insightful evidence for coexisting cues in natural contours, the factors were image features, and not necessarily perceptual quantities that represent the contours in the visual system. Electrophysiological studies have reported evidence of the cortical representation of angles and curvatures (Pasupathy and Connor, 1999; Ito and Komatsu, 2004), but not of other contour features such as convexity, closure, symmetry, etc. It is crucial to investigate whether these factors are indeed perceptual quantities that represent natural contours. The results of Fowlkes et al. (2007) could be biased because of the natural distribution of the appearance frequency of the factors. More frequently appearing factors could be judged more dominant than those appearing less frequently. The natural distribution could be biased so that contradictory cues barely coexist. It is desirable to annul the frequency of appearance of the factors when examining the effectiveness of coexisting factors. Investigations that focus on perceptual quantities using controlled natural contours are expected to provide crucial evidence for understanding figure–ground perception.

Figure–ground segregation is often considered as global processing that needs top-down processing. However, local bottom-up processing that is fast and autonomous should also contribute to the segregation before and recurrently with the top-down processing. For instance, local figure–ground assignment could differ from the global perception, depending on instruction, window size, or direction of gaze (Fowlkes et al., 2007; Kim and Feldman, 2009). Other psychophysical studies have reported that figure–ground segregation can occur without focal attention near the point of fixation (Kimchi and Peterson, 2008), and that human development shows differences in detecting local and global configurations (Kimchi et al., 2005). Physiological studies have reported that early- to intermediate-level visual areas respond to grouping and figure–ground segregation (e.g., Altmann et al., 2003; Tyler et al., 2005). A recent physiological study reported that figure–ground processing in the primary visual cortex (V1) follows each fixation saccade, indicating autonomous bottom-up processing of figure–ground segregation in an early stage (Gilad et al., 2014). Physiological studies of the cortical area (V2) have found a short latency of 20–50 ms for border-ownership-selective cells (e.g., Zhou et al., 2000; Zhang and von der Heydt, 2010). Although the origin of this latency is controversial, the contribution of bottom-up signals is plausible (Sakai and Nishimura, 2006; Super et al., 2010; Sakai et al., 2012; Sakai and Michii, 2013). Local, autonomous bottom-up processing appears to play a crucial role in figure–ground segregation.

We investigated whether the Gestalt factors, specifically convexity, closure, and symmetry, that coexist inherently in natural contours are perceptual quantities that represent local contours. We focused on these three factors as the first step because they appear to represent well the shape of a local contour segment. Specifically, we performed similarity tests between a variety of natural contour patches that were labeled by these three factors, and examined the contribution of the three factors to the perceptual similarity between the contours. The contour patches were sampled so that their distribution was uniform within the space composed of the three contour features. This sampling ensured the equal appearance frequency of the factors and a wide variety of contour shapes including those with contradictory factors that induce the figure in the opposite directions. Because the contradictory cases appear much less frequently in natural distribution, our sampling with uniform distribution is crucial for understanding the cue combinations. We used natural contours as a way to randomly choose a variety of contours that satisfy a wide range of cue combinations, but not to reflect the probability of natural appearance. Multidimensional scaling (MDS) analyses showed that combinations of convexity, closure, and symmetry contribute to the similarities of the natural contours and are indeed perceptual quantities.

**FIGURE 1:**
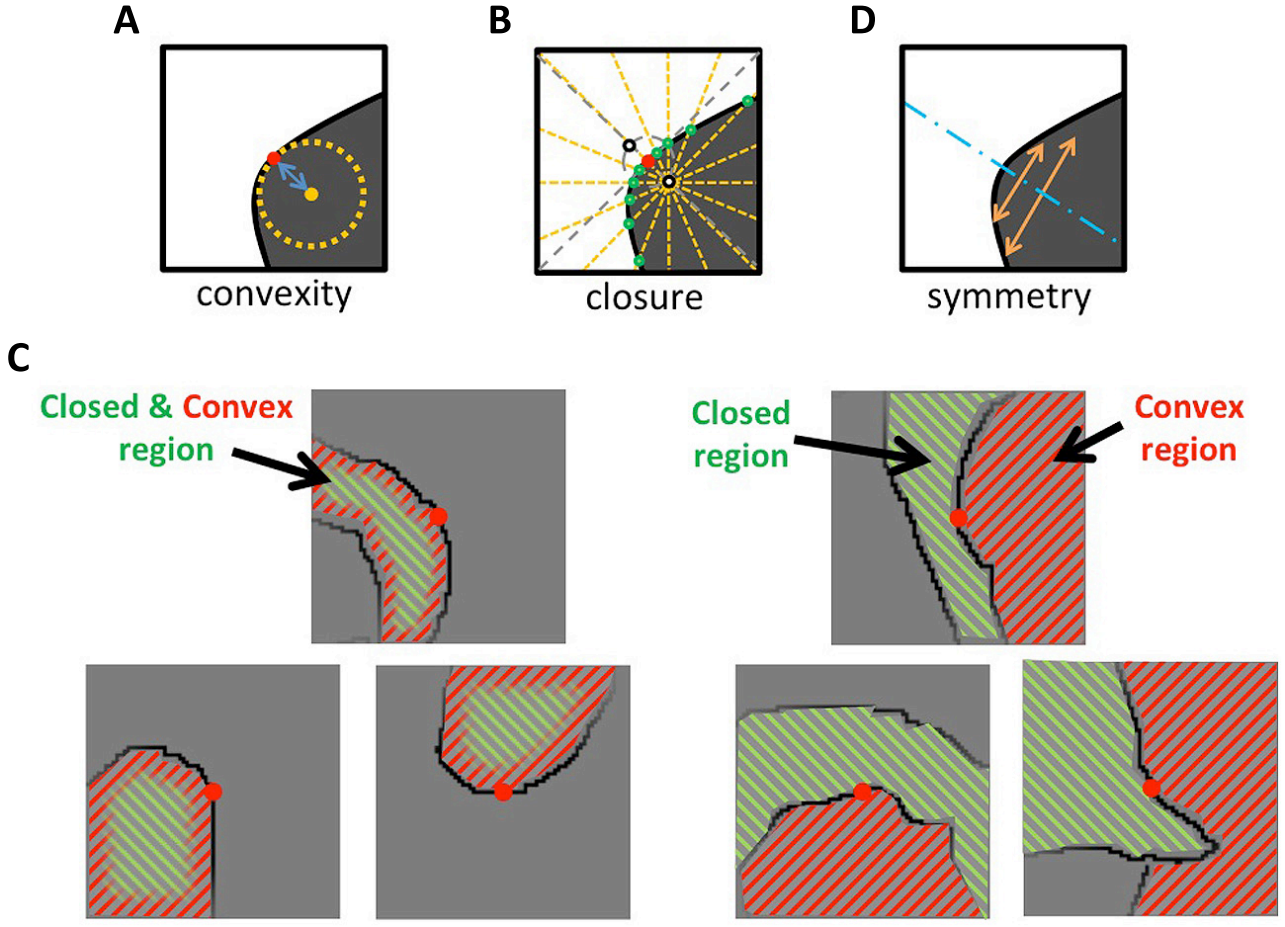
**Illustrations for the quantification of the three Gestalt cues. (A)** *Convexity* was defined as the curvature around the center of a patch. **(B)** *Closure* was defined from the number of radial lines that cross the contours. The degree of *Closure* is positive if a contour is closed more on the convex side, and negative if closed on the concave side. **(C)** The left panels show examples that *Closure* is positive where the convex side is closed more than the concave side (*N*_convex_ > *N*_concave_). In this case, convexity and closure draw figure–ground perception in the same direction. The right panels show examples that *Closure* is negative where the concave side is more closed. In this case, convexity and closure conflict with each other. **(D)** *Symmetry* was defined as the degree of overlap between the contours of both sides with respect to the symmetry axis. The blue dotted line shows the optimal symmetry axis. See the text for details.

Next, we examined whether convexity, closure, and symmetry contribute to local figure–ground segregation in the natural contours when they coexist with nearly equal probability. We performed psychophysical experiments to judge the direction of figure along the contours and examined the contribution of these factors to the figure–ground judgment. A multiple linear regression analysis (MLRA) indicated that closure was a significant factor but convexity and symmetry were not. This result indicates that closure is a stronger cue compared with convexity and symmetry for the figure–ground segregation in the local natural contours, which appears to be natural since convexity represents a part of closed contour. Because the degree of closure and convexity/concavity as well as their appearance frequency were controlled so that they distributed uniformly, these cues were contradictory in about a half of the stimuli. Our result suggests the dominance of closure over convexity when they are contradictory (see **Figure 1C** for examples). These results indicate that convexity, closure, and symmetry are perceptual quantities that represent local contours, and that closure is dominant among them when these factors coexist with equal probability including contradictory cases.

## Materials and Methods

To analyze the contribution of contour shape to the perception of similarity and local figure–ground segregation, we quantified contours with local cues, specifically, convexity, closure, and symmetry. In the present study, we used the standard definitions for convexity and closure (e.g., Sajda and Finkel, 1995; Fowlkes et al., 2007). Convexity was defined by curvature, and closure was defined by the number of radial lines that crossed the contour, as is shown in detail in the following subsections. Symmetry was defined as a quantitative measure that describes how close a local contour is to the axial symmetry (Sakai et al., 2014). We divided a number of natural contours into local patches, labeled them with the three measures, and sampled stimuli from them. We endeavored to sample patches so that they distributed uniformly over the space that was constituted of the three measures. This sampling resulted in a wide variety of contour shapes with a limited number of patches, including those with contradictory factors that induce figure in the opposite directions. Note that we used natural contours as a way to randomly pickup a variety of contours that satisfy particular cue combinations, but not to reflect natural appearance probability. However, the practical limitation on the number of the patches and the property of the natural distribution prevented us from collecting a complete set of patches with uniform distribution. Therefore, we sampled the patches uniformly from the space constituted of convexity and closure, and confirmed that symmetry of the chosen set was widely distributed. We used this set of local contours for the present experiments, with each patch labeled by the degree of convexity, closure, and symmetry. The experiments were approved by the Research Ethics Committee of the Institute of Systems and Information Engineering, University of Tsukuba, in accordance with the Code of Ethics of the World Medical Association (Declaration of Helsinki). Informed consent was obtained for experimentation with the participants.

### Definitions of Convexity, Closure, and Symmetry

We selected squares of 69 × 69 pixel from a set of contours that contained human-marked segmentation of natural scenes (Berkeley Figure/Ground Dataset, BFGD; Martin et al., 2001) with the center of a square always placed on the contour. The shape of the local contours was quantified with degrees of convexity, closure, and symmetry, as detailed below.

#### Convexity

**FIGURE 2:**
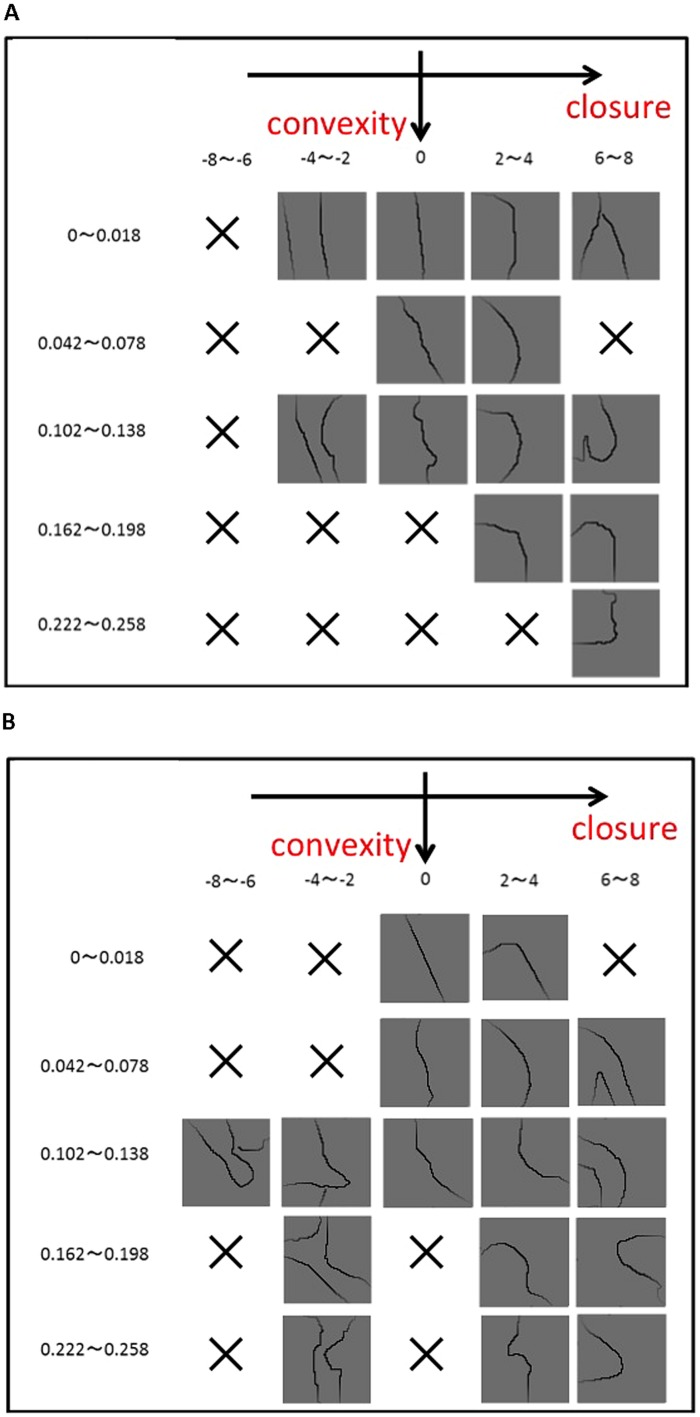
**Examples of the stimuli used in the experiments.** To ensure a wide variety of contour shape, we classified patches based on the degree of *Convexity*, *Closure*, and *Symmetry*, and sampled them as uniformly as possible. **(A)** The patches in which the contour tangent at the center ranges between 0° (vertical) and –15° (counterclockwise), and **(B)** between –15° and –30°. The degree of *Convexity* increases from the top (0–0.018) to the bottom (0.222–0.258), and *Closure* from the left (–8 to –6) to the right (6–8). There are a number of the patches in which *Convexity* and *Closure* correlate positively, but there are few or no patches with the negative correlation. Specifically, patches with a large *Convexity* and a negative *Closure*, as examples shown in **Figure 1C**, barely exist in the Berkeley Figure/Ground Dataset (BFGD).

We used the standard definition for convexity (Fowlkes et al., 2007) as defined by:

(1) $$Convexity\left(x_{u},\;y_{u}\right)=\log\left(1+\left|\rho \left(x_{u},\;y_{u}\right)\right|\right)$$

where ρ represents the curvature at the center of a patch (*x*_u_*, y*_u_). We took into account 41 pixels along the contour for the computation of ρ, with the midpoint at the center of a patch (ρ represents the curvature of a contour segment with the length of 41pixels). An illustration of *Convexity* is shown in **Figure 1A**. *Convexity* is always positive. The opposite of the convex side was defined as the concave side. Examples of patches with particular values of *Convexity* are shown in **Figure 2**.

#### Closure

The closure index, used in the present experiment, was defined by Sajda and Finkel (1995). In brief, closure was defined as the number of radial lines that crossed the contour in a square patch, as illustrated in **Figure 1B**. We considered the normal of the contour that passed through the center of a patch (*x*_u_*, y*_u_). We chose two points along the contour normal that were equidistant (5 pixel) from the center (black open circles in **Figure 1B**), and drew 16 radial lines with an increment of 22.5° from each of the two points. We counted the number of lines that crossed the contour for each point. *Closure* of a patch was defined as the difference between the number of crossing lines in convex side, *N*_convex_*_,_* and that in concave side, *N*_concave_:

(2) $$Closure\,\left(x_{u},y_{u}\right)=N_{convex}-N_{concave}\,$$

*Closure* is positive if the closed side corresponds with the convex side, and negative if it corresponds with the concave side. Examples of positive and negative cases are illustrated in **Figure 1C**. Examples of patches with particular values of *Closure* are shown in **Figure 2**.

#### Symmetry

We proposed the degree of symmetry as a quantitative measure to describe how close a local contour is to the axial symmetry (Sakai et al., 2014). Because contours are barely symmetric in terms of geometry, a measure to describe the degree of symmetry needs to be defined. The degree of symmetry was defined as the degree of the overlap of the contours between the two sides divided by the optimal axis of symmetry, as illustrated in **Figure 1D**. The optimal axis provides the maximum overlap of contours between the two sides, which was searched thoroughly by rotating (θ) and translating (*x*) the patch with respect to the vertical at the center. The overlap of the contours between the two sides (*a* and *b*) was given by:

(3) $${dos}_{\theta ,x}=\frac{\sum_{i=1}^{N}\left(\sum_{j=1}^{N}\left(a_{ij}b_{(N-i+1),j}\right)\right)}{length}\,$$

where *i* and *j* correspond to the *x* and *y* directions of the rotated/translated patch, respectively, with the axis of symmetry set to the vertical at the center and the origin at the top-left corner. *N* is the spatial extent of the patch in pixel (*N* = 69). The overlap was normalized by the length of contour in the patch (*length*). We normalized *dos* by the largest *dos* among all patches (*m*) taken from BFGD, and defined it as *Symmetry* for the patch *k*:

(4) $${Symmetry}_{k}=\frac{\max_{\theta ,x}\left({dos}_{k,\theta ,x}\right)}{\max_{m,\theta ,x}\left({dos}_{m,\theta ,x}\right)}\,$$

This normalization assures the independence of *Symmetry* from patch size (*Convexity* and *Closure* are independent, but not *dos*). We confirmed that the patch with the largest *dos* was perfectly symmetric, therefore, the patch with *Symmetry* = 1 shows perfect symmetry. The sign of *Symmetry* was set negative if the length of the optimal axis passed through the convex side was shorter than that through the opposite side. This sign setting establishes the consistency among convexity, closure, and symmetry, in terms of which side tends to be figure (the positive sides tend to be figure). For instance, if a region is convex and closed, and the symmetry axis passes through it, all the cues indicate consistently that this region is likely to be figure. By contrast, if the symmetry axis is located outside of the convex region, the convex and symmetry cues conflict with each other; convex draws the perception of the figure toward the inside of the convex region and symmetry toward the outside. In this case, the indices should have the opposite signs to clarify the direction that the index draws the figural perception.

### Stimulus Selection and Presentation

Based on the three indices, *Convex*, *Closure*, and *Symmetry*, we selected 105 patches from more than 10,000 patches taken from BFGD. Before the selection, we excluded patches that match at least one of the following conditions: (1) contours crossed each other (X-junction), (2) a whole object (e.g., human, animal, or flower) was visible, or (3) contours were packed (complicated contours were closely located to each other) so that it was difficult to assess the direction of figure. The second and third conditions were tested by the visual inspection of three people (two were the authors) who were familiar with the dataset and did not participate in the experiments.

To assure a wide variety of contours, we classified the patches based on *Convex*, *Closure*, *Symmetry*, and the orientation (tangent) of the contour passing through the patch center. The contour was classified into eight orientations (0–15°, 15–30°, 30–60°, 60–90°, 90–120°, 120–150°, 150–165°, and 165–180°; 0° = vertical). *Convex* and *Closure* were categorized into five classes, respectively, as indicated in **Figure 2**. The patches were grouped into 200 classes that consisted of five classes for *Convexity*, five for *Closure*, and eight for orientation. One patch was chosen randomly from each class. The limited number of patches and the natural distribution of contour shapes made it impossible to fill all the classes. We performed a preliminary experiment to test whether the rank orders of *Convexity* and *Closure* agreed with those of perception, and chose 105 patches that showed agreement. The details of this procedure are described in Appendix A in the Supplementary Material. The selected set of stimuli is shown in Appendix B in the Supplementary Material. We confirmed that *Symmetry* of the chosen patches was widely distributed. The distribution of the patches as a function of the factors is shown in Appendix C in the Supplementary Material. Although our measure of *Symmetry* reflects the perception in some degree, the agreement between the two was less accurate than those for convexity and closure, and the details are described in Appendix A in the Supplementary Material. Note that we were unable to obtain a complete set of patches with the uniform distribution of the three factors because of the practical limitation on the number of patches and the natural distribution of contour shapes. To smooth patch boundaries, we multiplied the patches with a Gaussian (σ = 17 pixel). Examples of the patches are shown in **Figure 2**. We presented the patches on a liquid crystal display (Mitsubishi RDT197S) that was placed in a dark room. Contours were presented in black (0.86 cd/m^2^) on a gray background (62 cd/m^2^). A fixation aid was presented in red (72 cd/m^2^).

## Perceptual Similarity of Local Contours – Experiment 1

We investigated whether convexity, closure, and symmetry are indeed perceptual quantities to represent the perception of contour shape. Although these factors are widely known as Gestalt factors for grouping and figure–ground organization, it is uncertain whether the factors are the perceptual quantities that represent the contour shape in natural scenes. We determined psychophysically the multidimensional configuration that represents the perceptual shape of local contour, and analyzed whether these factors could be the configuration axes. Specifically, we performed similarity tests between a wide variety of natural contour patches in which multiple cues coexist, and performed MDS analyses. We generated the multidimensional configuration that represented the perceptual distance of local contour shapes from the similarity tests, and examined whether this arrangement agreed with the configuration that was composed of *Convexity*, *Closure*, and *Symmetry*. If the result showed agreement, it suggests that these factors are indeed perceptual quantities in the judgment of the shape of natural contours.

### Procedure

**FIGURE 3:**
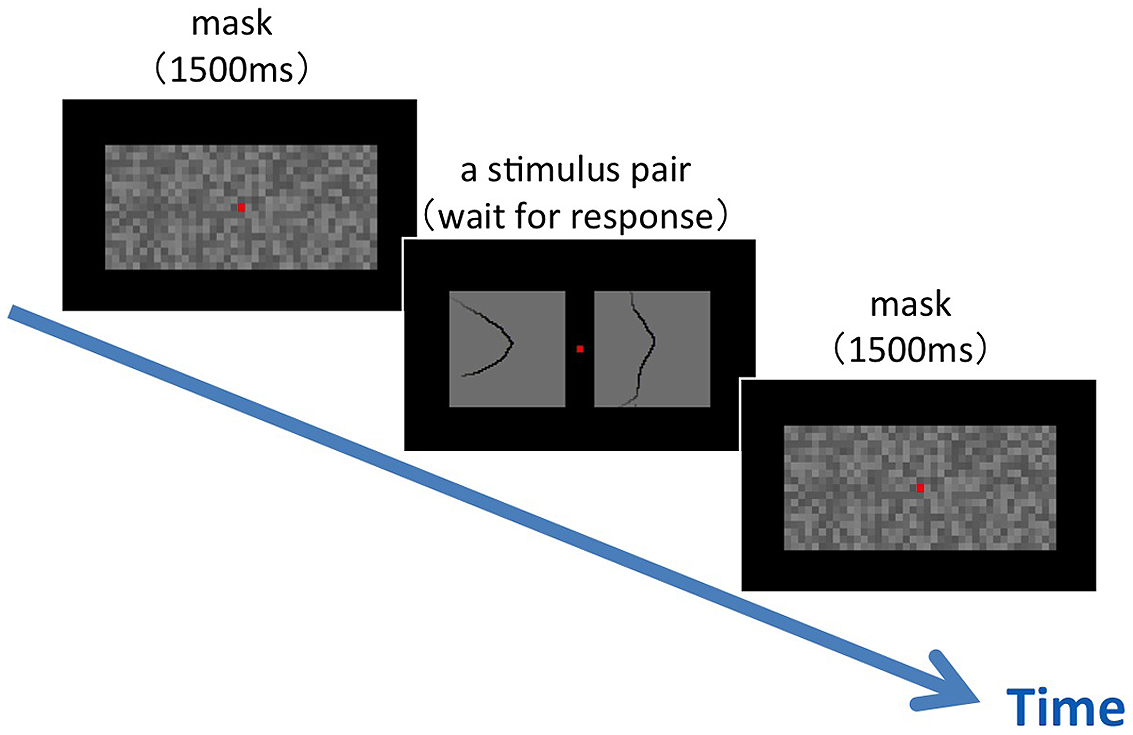
**An illustration of the experimental procedure for the similarity test.** Following a mask, a pair of stimulus patches was presented on the display. The participants were asked to judge the similarity between the two patches by the subjective rating method with five scales. A fixation aid, a small red square, was shown at the center of the display during the experiment.

We performed similarity tests between the natural contours that were labeled with *Convexity*, *Closure*, and *Symmetry*. To ensure a practical duration of the experiment, we limited the number of stimulus to 54 out of 105 (the selected set; see Materials and Methods). Specifically, we used the stimuli in which the contour tangent ranges between 0°–30° and 150°–180° with respect to the vertical. The experimental procedure is shown in **Figure 3**. Two stimulus patches of 4° × 4° in visual angle were presented side-by-side with the interval of 1° at the center of the display, following the presentation of a mask for 1500 ms. A fixation aid of a small red square was presented with the stimuli and the mask. Participants were asked to judge the similarity of the shape of the local contours in the right and left patches as soon as possible. Specifically, they were instructed to rate the similarity with five scales (subjective rating method) by choosing one key out of five. Immediately after the answer, the next mask was presented. Pairs of stimuli (*n* = 1431) were presented in random order with four repetitions. Six participants aged in their twenties (21–28) with normal or corrected-to-normal vision underwent the experiment. Before the experiment, the participants were familiarized with the task by performing the same procedure but with a small, different set of patches chosen exclusively from the selected set. During the familiarization, the participants were asked to categorize the patches evenly to five scales.

### Results

We performed tests between natural contours to determine the perceptual similarity among the contours. MDS was applied to generate the perceptual configuration of the contours that represents the similarity among patches by the distance between the points corresponding to the patches in a low-dimensional space (e.g., Dillon and Goldstein, 1984). In other words, perceptually similar contours were placed close to each other while dissimilar contours were placed separately at a distance in a space with one to three dimensions (1D to 3D). With this configuration, the rank order of perceptual similarity should agree with that of proximity. However, some patches violated this relation because the dimension of the configuration space was far lower than that of the patches. This error was evaluated by the *stress* as defined by Kruscal (1964) that takes zero for a perfect match between the rank orders and one for no match. The stress is defined as follows:

(5) $$stress=\frac{{\Sigma \Sigma \left[\theta \left(d_{ij}\right)-{\overset{\wedge }{d}}_{ij}\right]}^{2}}{\Sigma \Sigma {\overset{\wedge }{d}}_{ij}^{2}}\,$$

where θ is a monotonically increasing function (rank order), and *d* and 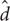 represent the Euclidian distances between stimuli *i* and *j* in perceptual and topographical configurations, respectively. We applied the Kruscal (1964) method (isoMDS in R language for statistical computing) to compute the stress in which the absolute value of the similarity rating was ignored. The mean stresses for 1D, 2D, and 3D configurations among all participants were 43, 24, and 18%, respectively. The details are shown in Appendix D in the Supplementary Material. This result suggests that the correctness for the 1D configuration in the representation of perceptual similarity is about half of the perfect, and that the correctness increases to more than 80% for the 3D configuration. These stress values indicate the limitation of the analysis by reducing the dimension. Specifically, 1D, 2D, and 3D analyses explain no more than 57, 76, and 82% of the perceptual similarity, respectively.

We examined whether topographical measures, *Convexity, Closure*, and *Symmetry*, account for the perceptual configuration. Specifically, we tested whether the perceptual configuration of the contours agrees with the topographical configuration of the stimuli. In other words, we generated topographical configurations of the contours by mapping the patches into 1D to 3D spaces as defined by the topographical measures and compared them with the perceptual configurations of the same dimension. If the perceptual and topographical configurations show agreement, it suggests that the topographical factors are perceptual quantities in the judgment of similarity between the contours. The agreement further suggests that *Convexity, Closure*, and *Symmetry* account for the perception of the contours.

**FIGURE 4:**
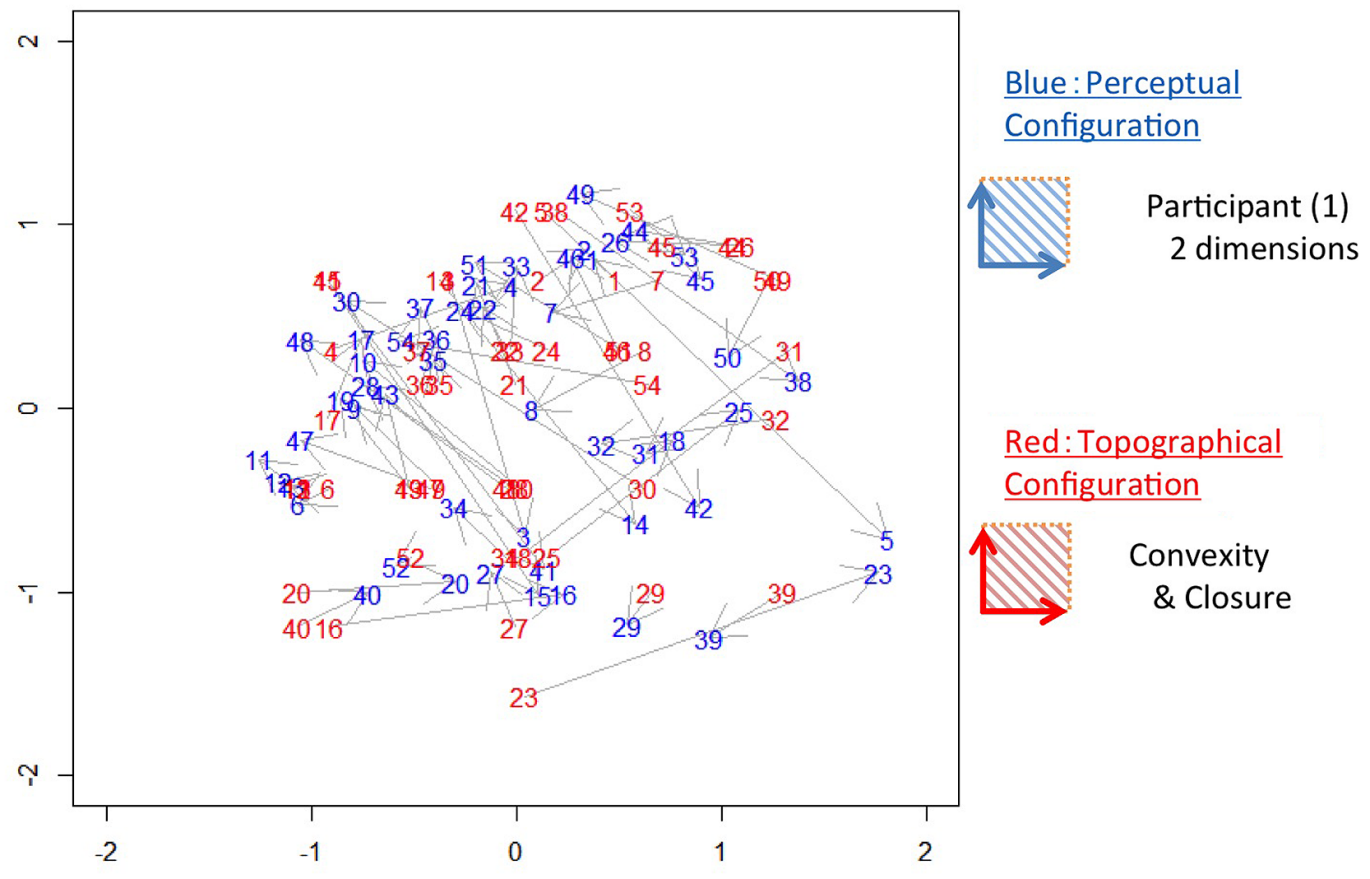
**An example of the perceptual and topographical configurations.** Numbers in blue (dark gray) represent stimuli in the 2D perceptual configuration in which shorter distances between stimuli indicate greater perceptual similarity. Numbers in red (light gray) represent stimuli in the topographical configuration of *Convexity* and *Closure*. The distances between the corresponding stimuli (the same numbers in red and blue) indicate the similarity between the perceptual and topographical configurations.

Before the comparison of the perceptual and topographical configurations, we processed the configurations so as to minimize the differences in arrangement between the configurations (Borg and Groenen, 1997). Specifically, we scaled and rotated the configurations using the Procrustes method (Hurley and Cattell, 1962; *procrustes* function in Vegan package of R) to obtain the best fit (the minimum difference). An example of the configurations of perception and topography with best fit (*Convexity* and *Closure*) in 2D is shown in **Figure 4**. The 2D perceptual configuration has two axes, which means that contour shapes are labeled with two factors. Here, we examined whether the contour shape is represented perceptually by convexity and closure. Some patches are located close to each other between the two configurations (the same numbers in red and blue in **Figure 4**; e.g., Nos. 1, 29, and 40), while others are distant (e.g., Nos. 5, 23, and 42).

To test the significance of the agreement between the perceptual and topographical configurations, we used the squared sum of the pair-wise Euclidian distance of the patches between the configurations (Borg and Groenen, 1997). We defined this squared-sum of distance as *Error*. To enable intuitive comparison, we normalized the *Error* by the squared-sum of the distance between the perceptual and random configurations (*Error* for random configurations). The random configurations were computed repeatedly 100 times by scrambling the similarity among the patches. For the example shown in **Figure 4**, the mean *Error* among all participants was 0.57, indicating that *Convexity* and *Closure* account for 43% of the perceptual similarity. A statistical test showed that the *Error* was significantly smaller than that for the random configurations (*t*-test, *p* < 0.001). This result indicates an agreement between the perceptual and topographical configurations, suggesting that *Convexity* and *Closure* contribute to the perceptual similarity between natural contours.

**FIGURE 5:**
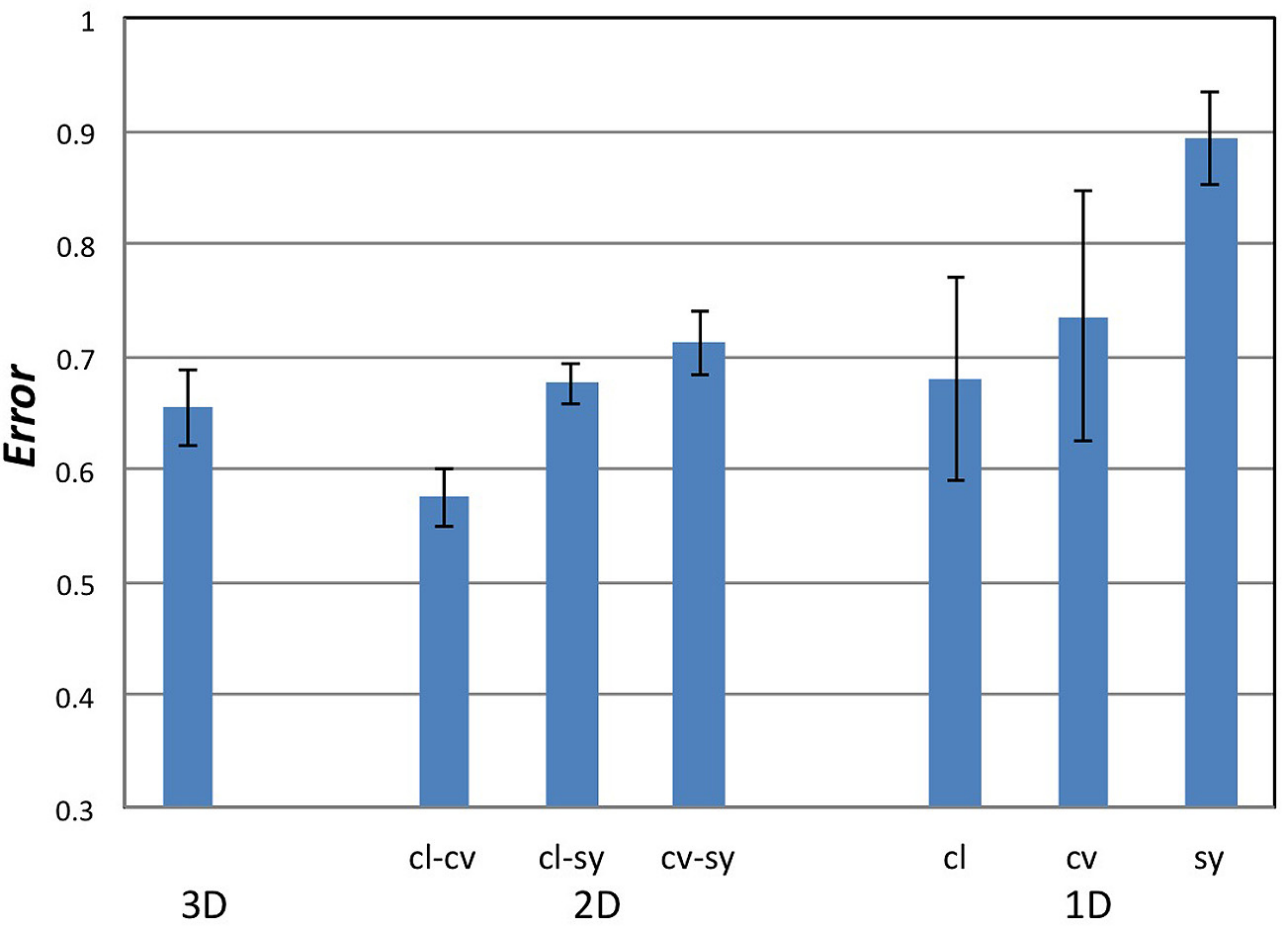
**The magnitudes of *Error* in the MDS analyses. cl, cv, and sy stand for *Closure, Convexity*, and *Symmetry*, respectively.** The combination of *Closure* and *Convexity* in 2D configuration showed the lowest *Error* of 57%, indicating that *Closure–Convexity* accounts for 43% of the similarity among the local contours.

We performed the analyses for 1D to 3D configurations in which *Convexity*, *Closure*, and *Symmetry* were topographical factors. The computed *Errors* and their statistical tests are summarized in **Figure 5** and **Table 1**, respectively, and their details are shown in Appendix D in the Supplementary Material. For the comparison of 1D configurations between the perceptual and topographical configurations, five of six participants found significantly smaller *Error* than that for the random configurations (*t*-test, *p* < 0.05) for *Closure*, and three for *Convexity* or *Symmetry*. The mean *Errors* among participants for *Closure, Convexity*, and *Symmetry* were 68, 74, and 89%, respectively. These results indicate that *Closure, Convexity*, and *Symmetry* account for about 10–30% of the similarity. The contribution of *Closure* was significantly larger than *Symmetry* but not than *Convexity* (pair-wise *t*-test; *p* = 0.01 and 0.3, respectively). The contribution of *Closure* and *Convexity* are surprisingly high. Because the stress of 43% indicates that 1D configuration represents no more than 57% of the perceptual similarity, *Closure* and *Convexity* account for about a half of the similarity.

**Table 1:** The results of multidimensional scaling (MDS; Experiment 1) for the 1D to 3D configurations in which *Convexity*, *Closure*, and *Symmetry* were topographical factors.

1 dimension	2 dimensions	3 dimensions
Convexity 3/6	Convexity and Closure 6/6	Convexity and Closure and Symmetry 6/6 participants were significant
Closure 5/6	Closure and Symmetry 6/6	
Symmetry 3/6	Closure and Convexity 6/6	

*The table shows the number of participants whose perceptual configuration was significantly different (p < 0.05) from the random configurations. All participants showed significance in all 2D and 3D combinations.*

For the 2D and 3D configurations, all six participants found significantly smaller *Error* than that for the random configurations (*t*-test, *p* < 0.0013) for all combinations of factors, indicating that these factors contribute to the perceptual similarity between natural contours. For 2D configurations, the combination of *Closure–Convexity* showed the lowest mean *Error* of 57%, indicating that *Closure*–*Convexity* accounts for about 43% of the similarity. The mean *Errors* for *Closure–Symmetry* and *Convex–Symmetry* were 68 and 71%, respectively, indicating that *Closure–Symmetry* and *Convex-Symmetry* account for about 32 and 29% of the similarity, respectively. The contributions of these two combinations were significantly smaller than that of *Closure-Convexity* (pair-wise *t*-test, *p* < 0.01). This result suggests that *Closure* and *Convexity* work independently in some cases, although these factors are moderately dependent in topography [Pearson correlation: γ = 0.42 (*p* < 0.01)]. The mean *Error* for the 3D configuration was 65%, indicating that the combination of the three factors account for 35% of the similarity. Although this contribution is lower than that of *Closure–Convexity* in 2D (43%; pair-wise *t*-test, *p* < 0.01), two participants showed greater contributions in the 3D case compared to the 1D and 2D cases. The combination of the three factors contributes less than a half of the perceptual similarity, suggesting the involvement of other factors in the perception of contour segments. This result appears natural because contour similarity may also be judged by other factors besides the three factors examined here. These results suggest that *Convexity*, *Closure*, and *Symmetry* are perceptual quantities that represent the local shapes of natural contours. *Closure* and *Convexity* showed greater contribution than *Symmetry* in the representation of similarity with an indication of being independent from each other. It should be noticed that the correlation between *Symmetry* and the perception was lower than that for *Convexity* and *Closure*. Although a quantity that better describes the perception of symmetry for arbitrary contours has not been proposed, a further study with such quantity will further reveal the contribution of symmetry.

## Figure–Ground Segregation in Local Contours – Experiment 2

We examined whether convexity, closure, and symmetry contribute to local figure–ground segregation in natural contours. We performed psychophysical experiments to judge the direction of the figure along natural contours, and examined the contribution of these factors to the figure–ground judgment.

### Procedure

**FIGURE 6:**
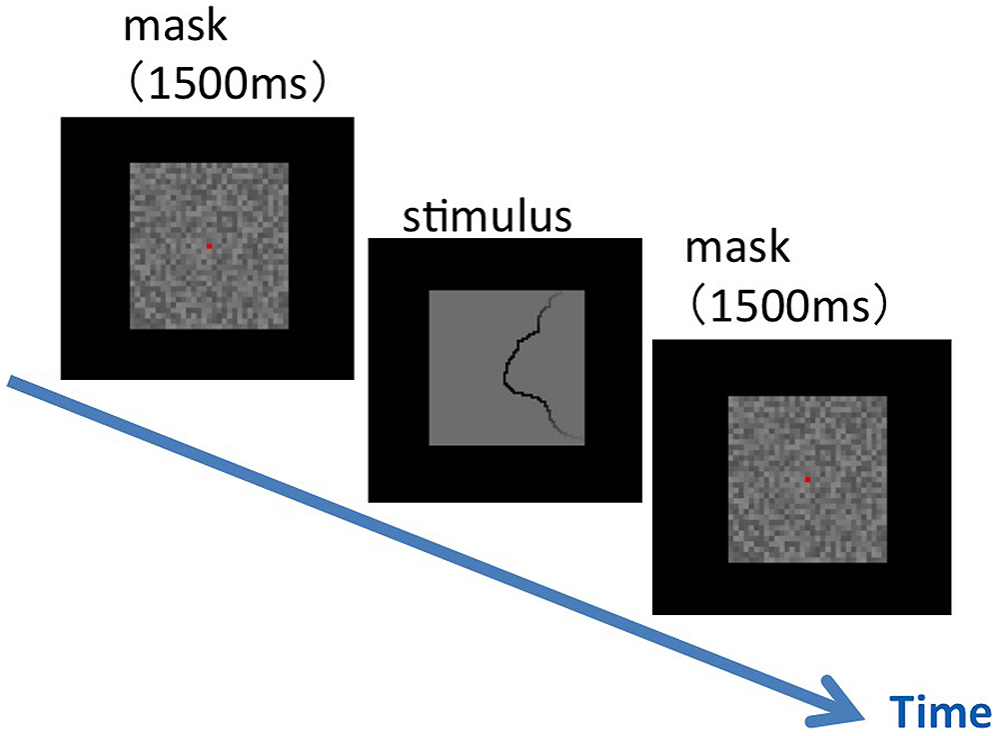
**An illustration of the experimental procedure for the figure–ground judgment.** Following a mask, a single stimulus patch was presented on the display. The participants were asked to judge as soon as possible the direction of the figure at the patch center that was indicated by a fixation aid (a small red square shown with a mask).

We presented the selected set of 105 patches of natural contours (see Materials and Methods), and measured the direction of the figure along the contours. The experimental procedure is shown in **Figure 6**. We divided the set of patches into two groups, vertical and horizontal groups, depending on the orientation of the contour (0 ± 45° and 90 ± 45° for vertical and horizontal groups, respectively). Patches from either group were presented for a single session. A single stimulus patch of 4° × 4° in visual angle was presented at the center of the display, following the presentation of a mask for 1500 ms. A fixation aid of a small red square was presented with the mask. Participants were asked to judge the direction of the figure at the patch center, the location of the fixation aid, by pressing an assigned key in a two-alternative forced-choice paradigm. Specifically, participants were asked to answer right or left for the vertical group, and top or bottom for the horizontal group. Immediately after the response, a mask was presented, and the next trial was begun. All patches were shown in random order with the repetition of 100. Six participants aged in their twenties (21–28) with normal or corrected-to-normal vision underwent the experiment.

### Results

**FIGURE 7:**
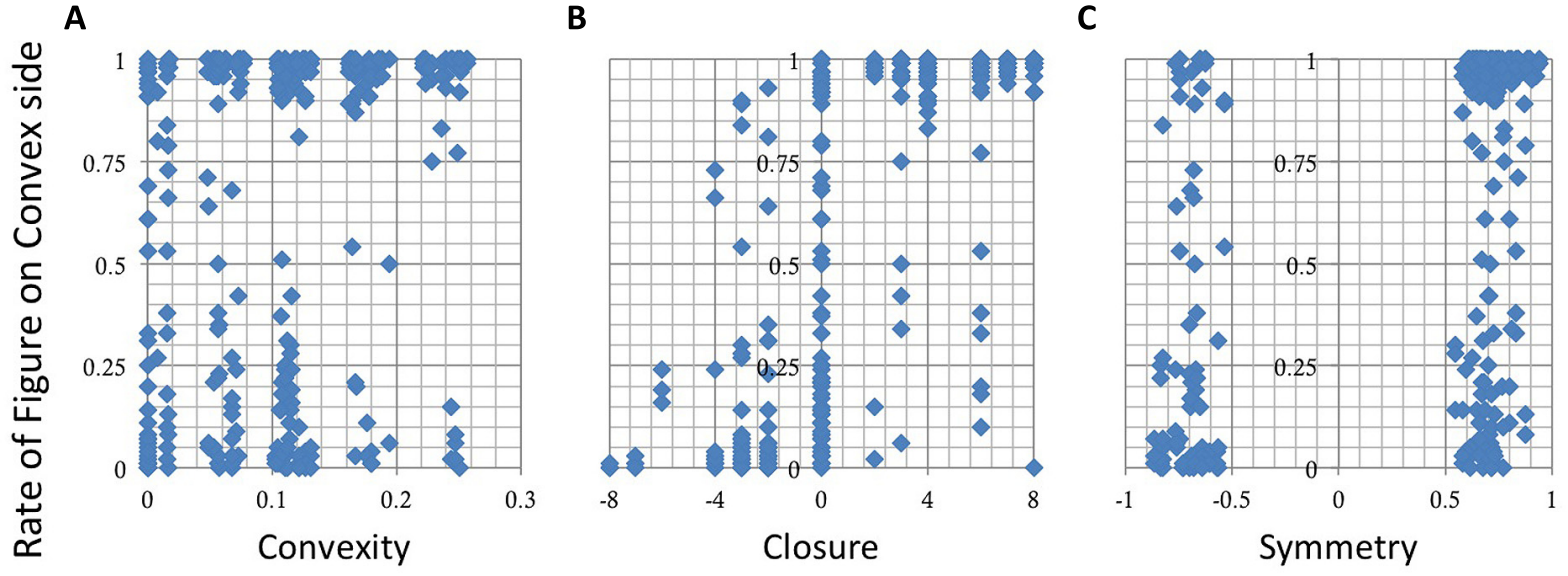
**The results of the figure–ground judgment.** The rate of choosing a convex side as figure is shown as a function of *Convexity* **(A)**, *Closure* **(B),** and *Symmetry* **(C)**. The responses depend on *Closure* but not on *Convexity* or *Symmetry*.

We plotted the rate of choosing a convex side as a function of the indices, *Convexity*, *Closure*, and *Symmetry*. The overall data from all participants are shown in **Figure 7**. The result shows that the responses depend on *Closure* (**Figure 7B**) but not on *Convex* and *Symmetry* (**Figures 7A,C**). We plotted the ratio of judgment in which the participants perceived figure on the convex side, thus a positive *Closure* means that the *convex* and closed side was chosen, and a negative means the *concave* and closed side. Therefore the results indicate that a closed side was perceived as a figure independent of convexity (**Figure 7B**). A MLRA showed that *Closure* is significant, but *Convexity*, *Symmetry*, and their interactions are not, as shown by the results summarized in **Table 2**. The adjusted coefficient of determination, *R*^2^ was 0.54, indicating a reasonable fit. These results indicate that closure is dominant among the three factors for figure–ground perception in local contours when convexity, closure, and symmetry coexist with equal probability including contradictory cases.

**Table 2:** The results of multiple linear regression analysis (MLRA) in Experiment 2.

	Regression coefficient	*p*-value
(Intercept)	0.490	<2e-16^∗∗∗^
Convexity	-0.334	0.262
Closure	0.0755	6.14e–13^∗∗∗^
Symmetry	0.0383	0.446
Convexity × Closure	-0.0200	0.772
Convexity × Symmetry	0.282	0.510
Closure × Symmetry	0.00557	0.536
Adjusted *R*^2^: 0.537		

*Closure was significant, but Convexity, Symmetry, or interactions were not. The adjusted coefficient of determination, R^2^ was 0.54, indicating a reasonable fit. The result indicates that closure is a dominant cue for figure–ground perception in natural contours. ^∗∗∗^p < 0.01.*

*The model:*

*(Rate of BO on Convex side) = a + b ⋅ Convexity + c ⋅ Closure + d ⋅ Symmetry + e ⋅ Conv × Clos + f ⋅ Conv × Sym + g ⋅ Clos × Sym*

The present result did not show significance in symmetry. A possible bias could be evoked by fixation. We instructed participants to fixate at the patch center where the contour (that defines closure and convexity) always presented while the symmetry axis might not. To examine this possibility, we conducted the same procedure without the fixation. We observed no significant difference from the results with the fixation presented above, indicating no effect of the fixation.

## Discussion

We investigated the perceptual representation of local contour shapes and the contribution of them to local figure–ground segregation. We quantified contour shapes by local cues: convexity, closure, and symmetry. To ensure a wide variety of contour shapes, we sampled patches from natural scenes so that they distributed uniformly over the space that was composed of the three factors. First, we investigated whether the three factors are indeed perceptual quantities that represent the contour shapes. We performed similarity tests between the contour patches, and examined the contribution of the factors to the perceptual similarity between the natural contours. MDS analyses showed that combinations of convexity, closure, and symmetry contribute to the similarity, and thus these factors are indeed perceptual quantities. Second, we examined whether the three factors contribute to the perception of local figure–ground. We performed psychophysical experiments in which participants judged the direction of the figure along the natural contours, and examined the contribution of the factors to the figure–ground judgment. MLRA showed that closure reached significance, but convexity and symmetry did not. These results indicate that closure is a dominant cue for local figure–ground perception when convexity, closure, and symmetry coexist with equal probability including contradictory cases.

Convexity has long been known as a strong cue for figure–ground perception. Combinations with other cues have also been studied, including the comparison with symmetry (e.g., Kanizsa and Gerbino, 1976; Devinck and Spillmann, 2013; Mojica and Peterson, 2014), parallelism (Froyen et al., 2013), and top-down effects (Devinck and Spillmann, 2013). These studies showed relative strength of convexity compared with the other cues. For instance, convexity evoked stronger 3D volume from structure-from-motion compared with symmetry and parallelism. Convexity evoked shorter reaction times for detecting figures in ambiguous boundaries compared with symmetry and top-down effects. However, these studies presented multiple, artificial contours with simple and regular shapes in which two cues were contradictory. Our experiments were distinct from these previous studies in stimuli; (1) ours consisted of a single contour in a number of cases, and (2) our stimuli were controlled natural contours in which multiple cues coexist with a wide range of relative strengths from consistent to contradictory cases. Fowlkes et al. (2007) used natural contours to test the relative strength of convexity, relative size, and lower region, and reported that convexity was weakest among the three cues. Natural contours in which multiple cues are inherent in a single contour may work differently from pairs of artificial contours with contradictory cues.

Although our results did not indicate the significance of convexity, our result may be consistent with previous reports. Gestalt factors are not necessarily independent. Convexity and closure appear to be often dependent on each other; in fact, the coefficient of Pearson correlation between *Convexity* and *Closure* was 0.42 (*p* < 0.01) in our stimulus set. A closed contour within a stimulus patch has a large degree of convexity. Thus, the essence of convexity can be considered closedness. From an ecological viewpoint, a closed contour is a strong cue for an object (figure). If the object is occluded and only some parts of the object are visible, convexity may be a good cue to substitute for closure. Convexity and closure can also be independent. As shown in the right panels of **Figure 1C**, a closed region may have a concave contour. Our result showed that in such a case, closure is dominant over convexity in figure–ground perception. Because we designed the experiments to have a wide and uniform distribution of the indices of the stimulus set, the number of the independent cases was close to that of the dependent cases. This uniform distribution is different from the natural distribution in which the dependent cases are more frequent. The uniform distribution appears to successfully separate the indices. If a stimulus set had the natural distribution, a greater number of dependent cases might conceal the independent cases and result in a failure to separate the indices.

Our results did not indicate the significance of symmetry in the figure–ground judgment. Symmetry has long been known as a strong cue for the perception of an object, and also as a basis for the neural representation of shape (e.g., Hung et al., 2012; Hatori and Sakai, 2014). The adaptation to symmetry has recently reported, indicating that symmetry is a perceptual quantity (Gheorghiu et al., 2014; Sakai et al., 2014). A crucial difference between our experiment and the previous studies is that our stimuli were local contours that consisted mostly of a single open contour. Thus, our results suggest that symmetry may not be as influential as closure with an open local contour. Symmetry may be crucial from an ecological viewpoint, such as in the detection of a living being that often has a closed contour.

## Conflict of Interest Statement

The authors declare that the research was conducted in the absence of any commercial or financial relationships that could be construed as a potential conflict of interest.
