# Supplementary material for: Perceptual representation and effectiveness of local figure–ground cues in natural contours
Source: Front Psychol. 2015 Nov 3;6:1685. doi: 10.3389/fpsyg.2015.01685 (PMC4630503; doi:10.3389/fpsyg.2015.01685)
Supplement: Supplementary file 1 [file Data_Sheet_1.PDF]

Appendix A

We performed a preliminary experiment to test whether the rank orders of *Convexity* and *Closure* agreed with those of perception. We divided the stimuli (114 patches) into ten sets (11 or 12 patches for each set) by randomly choosing two or three patches from each of the five *Convexity* classes. A single stimulus set was presented simultaneously, and the participants were asked to rank the convexity of the patches. We repeated this test three times. The mean rank of convexity for the six participants was computed and compared with the rank of *Convexity*. We performed the same tests with closure. The perceptual ranks and the computed ranks agreed for most patches; however, for 11 patches, the ranks did not match. We excluded the contradictory patches from the stimulus set and included new patches that were chosen randomly from the same class if there was another patch in that class. This procedure was repeated until all patches were in agreement (three repeats). We obtained a set of 105 patches in which the rank orders of *Convexity* and *Closure* agreed with those of the perception. Although this procedure does not provide the complete rank order of 105 patches, we used this procedure because the simultaneous comparison of 105 patches was not realistic in terms of time and difficulty in task. The selected set is shown in Appendix B.

To determine the perceptual rank of symmetry, we performed the same preliminary experiment with the selected set of stimulus (105 patches). In this experiment, we asked participants to judge symmetry rather than convexity and closure. Pearson's correlation coefficient between the perceptual rank-order and the value of *Symmetry* was 0.47. The correspondence of *Symmetry* with perception was less accurate than those with *Convexity* and *Closure*. It did not escape from our attention that the perceptual symmetry axis varied among participants. We examined how much the perceptual symmetry axis agrees with the axis determined during our computation of *Symmetry*. The mean of agreement among all stimuli and participants was 55%, with a wide range of distribution. However, good agreement was observed for stimuli that evoked consistent perception among participants (80% match for the stimuli with 60% consistency). We also found that stimuli with a relatively large magnitude of *Symmetry* evoked good agreement with perception. Details of these findings were reported by Sakai, *et al.* (2014). The results indicate that our measure of symmetry reflects perception to some degree, although less accurately than convexity and closure where the rank order of computation and perception match closely.

Appendix B

The set of local contour patches that were sampled from natural contours (the selected set). The patches used in Experiment 1 were indicated by green lines, with the numbers indicating the orientation of contour at the patch center. The contour patches were sampled so that their distribution was uniform in the space composed of the three contour features. This sampling ensured the equal appearance frequency of the factors and a wide variety of contour shapes including those with contradictory factors that induce figure in the opposite directions.

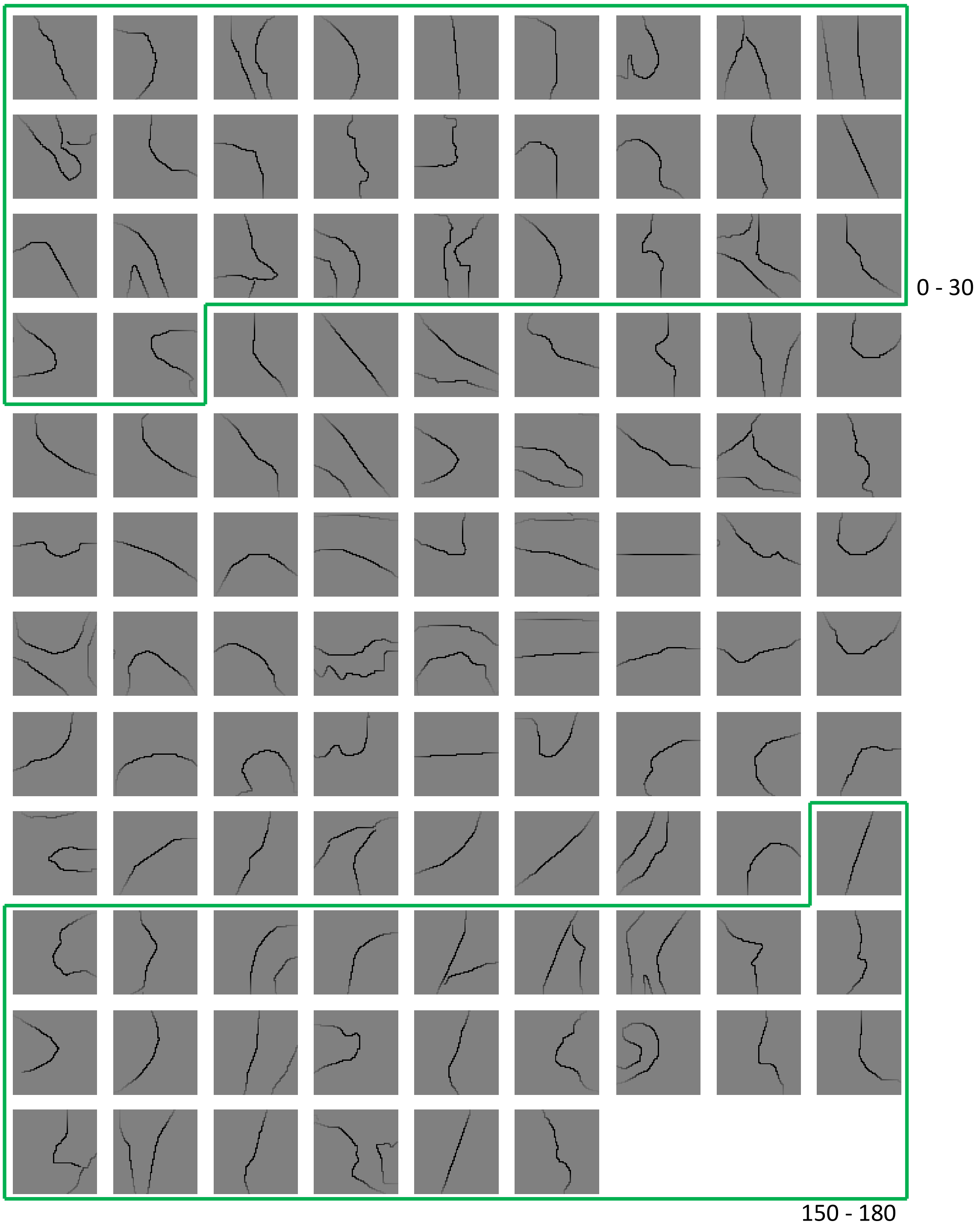

Appendix C

This appendix shows the distribution of *Convexity*, *Closure,* and *Symmetry* for the stimulus patches used in Experiments 1 and 2 (the middle and top rows, respectively). In all cases, the distributions do not show clear unbalance. Note that certain combinations of cues rarely or never exist in the patches of natural contours, such as patches with high convexity and low closure (i.e., the convex side is less closed than the concave side) and patches with high closure and low symmetry (i.e., the symmetry axis barely passes through a closed region). We chose the range of *Symmetry* between 0.5 and 1.0 because by definition, patches with small values do not appear symmetric (Sakai *et al.*, 2014). Examples of patches with their value of *Symmetry* are shown in the bottom row. The coefficient of Pearson correlation between *Convexity* and *Closure* was 0.42 ($p<0.01$), and that between Symmetry and Convexity/Closure were less than 0.2 ($p>0.01$).

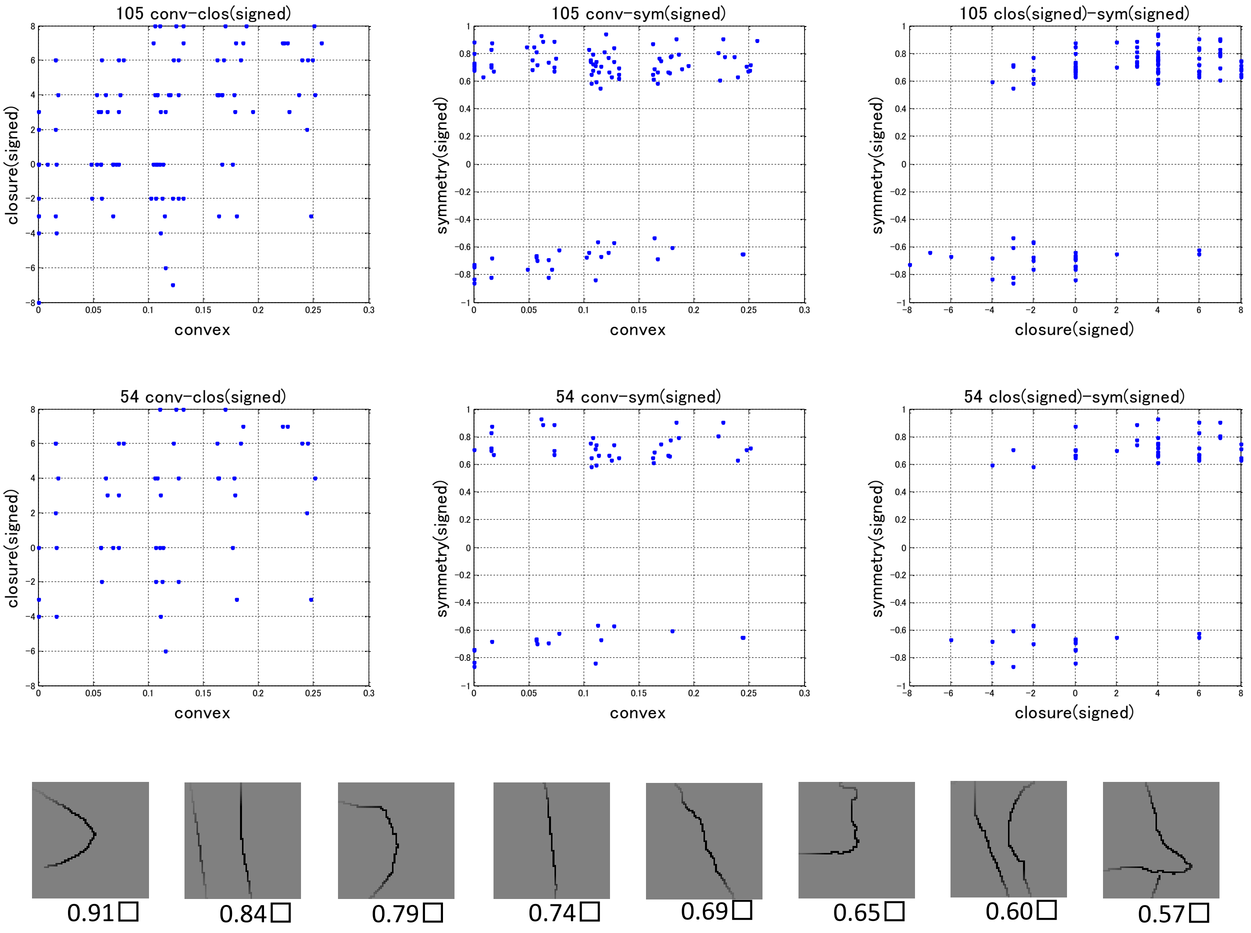

Appendix D

This appendix shows the individual results of Experiment 1. The tables show the stress and the normalized *Errors* with the *p* values of *t*-test for 3D, 2D, and 1D configurations. The mean stress decreases as the dimension increases, but the stress was still 17.9 for 3D configuration. For 2D and 3D configurations, the statistical tests with respect to the random configurations showed very low *p* values (<0.0013), indicating the significance of the three factors for the perception of contours. The number of asterisks indicates the significance of *p* value: * for <0.05 and *** for <0.01.

3D

Participant	Error Index	P value	
1	0.619	0.0000	***
2	0.751	0.0000	***
3	0.760	0.0001	***
4	0.584	0.0000	***
5	0.579	0.0000	***
6	0.629	0.0000	***
mean	0.654		

Stress

Participant	3D	2D	1D
1	16.5	22.4	35.1
2	14.2	20.5	39.3
3	18.8	24.1	43.4
4	17.7	22.7	38.9
5	19.0	25.6	49.7
6	21.2	26.6	50.5
mean	17.9	23.7	42.8

2D convex-closure

Participant	Error Index	P value	
1	0.541	0.0000	***
2	0.603	0.0000	***
3	0.659	0.0000	***
4	0.584	0.0000	***
5	0.474	0.0000	***
6	0.585	0.0000	***
mean	0.574		

1D convex

Participant	Error Index	P value	
1	0.359	0.000	***
2	0.964	0.330	
3	0.928	0.188	
4	0.753	0.001	***
5	0.442	0.000	***
6	0.965	0.333	
mean	0.735		

2D convex-symmetry

Participant	Error Index	P value	
1	0.597	0.0000	***
2	0.690	0.0000	***
3	0.797	0.0013	***
4	0.736	0.0000	***
5	0.691	0.0000	***
6	0.761	0.0002	***
mean	0.712		

1D closure

Participant	Error Index	P value	
1	0.572	0.000	***
2	1.035	0.663	
3	0.455	0.000	***
4	0.521	0.000	***
5	0.645	0.000	***
6	0.854	0.039	*
mean	0.680		

2D closure-symmetry

Participant	Error Index	P value	
1	0.681	0.0000	***
2	0.626	0.0000	***
3	0.650	0.0000	***
4	0.724	0.0000	***
5	0.639	0.0000	***
6	0.734	0.0001	***
mean	0.676		

1D symmetry

Participant	Error Index	P value	
1	0.809	0.010	***
2	1.005	0.524	
3	0.886	0.081	
4	0.822	0.014	*
5	0.804	0.008	***
6	1.036	0.669	
mean	0.894		
